# Methods for Casting Subterranean Ant Nests

**DOI:** 10.1673/031.010.8801

**Published:** 2010-07-02

**Authors:** Walter R. Tschinkel

**Affiliations:** Department of Biological Science, Florida State University, Tallahassee, FL 32306-4370

**Keywords:** charcoal, dental plaster, Formicidae, melting aluminum, melting zinc, nest architecture

## Abstract

The study of subterranean ant nests has been impeded by the difficulty of rendering their structures in visible form. Here, several different casting materials are shown to make perfect casts of the underground nests of ants. Each material (dental plaster, paraffin wax, aluminum, zinc) has advantages and limitations, which are discussed. Some of the materials allow the recovery of the ants entombed in the casts, allowing a census of the ants to be connected with features of their nest architecture. The necessary equipment and procedures are described in the hope that more researchers will study this very important aspect of ant natural history.

## Introduction

Scientific fields flower when effective methods of work become available. Most scientific advances, from morphology to molecular biology to nuclear physics, have been driven by the availability of new methods or instruments. In the study of social insects, nests constructed by wasps and bees are readily seen and studied, but the excavated subterranean nests of ants are invisible from the ground surface and are difficult to render. Yet, a large proportion of ant species nest in the ground, with the result that one of their most important activities, nesting, is not easily amenable to study. Most past studies have relied on sketches and measurements made during nest excavations to visualize the nest (see Tschinkel 2004 for a review), but these methods have great limitations.

More recently, the making of casts of subterranean nests has been shown to create a perfect replica of the hollow space that composes the nest (Mikheyev and Tschinkel 2004; Tschinkel 2003, 2004, 2005). Since Williams and Lofgren (1988) introduced the use of dental plaster for nest casting, I have used it and several other materials, including molten aluminum, molten zinc, and paraffin wax. Each material has advantages and disadvantages. The present paper describes the methods and equipment developed for each of these casting materials, their advantages and limitations, and examples of nest casts made with each. If others begin to make nest casts, perhaps the study of subterranean ant nests will flower too.

## Methods and Materials

### Choice of casting material

**Figure 1.**
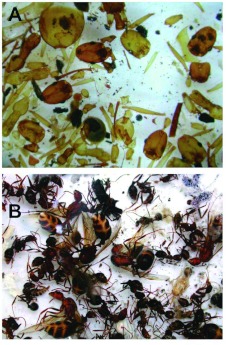
(A) Pieces of *Aphaenogaster floridana* workers recovered from a dental plaster cast dissolved in warm water over a period of about 4–5 weeks. Ants occasionally survive this process in one piece but usually break up as seen here. A head count serves as a census. Alternately, one can count legs and divide by six. (B) Ants recovered by melting of a cast made from paraffin wax. All the ants, including the brood, are intact and can be used for further study. High quality figures and videos are available online.

**Dental plaster.** The advantages of dental plaster are that it is cheap, easy to use, requires little equipment, and makes a fine cast. Its disadvantages are that it does not effectively cast nests with very narrow shafts and that the cast always breaks into pieces upon excavation, sometimes many pieces. The pieces must therefore be removed to the laboratory, dried and glued together with 5-min epoxy cement, a process that can be tedious as the pieces are numerous. Another advantage of plaster is that, when the reassembled cast has been studied and photographed, it can be broken apart, the pieces placed into fine mesh bags in running water, and the plaster dissolved away. The process takes 4–6 weeks, and when complete, allows the recovery of the ants entombed in the plaster (Figure 1A). The ant census is obviously an important datum in the study of nest architecture. Moreover, certain additional measures, such as the sizes and size-distribution of the ants are also possible. Unfortunately, the ants are usually broken into pieces, and few brood are recovered.

With plaster, as with paraffin wax (see below) the casting material captures most of the ants in their location at the time of casting, because the casting material enters chambers from the shaft, sweeping chamber contents toward the outer perimeter of the chambers. The same is true for metal casting, where the charred bodies of the ants can be seen along the edges of the cast chambers. The exceptions would be any ants that happened to be in the vertical shafts at the time of casting, but they would be a minor portion of the colony.

Several brands of orthodontic plaster are available from dental supply companies. Labstone (www.heraeus-dental-us.com/en/ourproducts/laboratory_1/modernmaterials/modernmaterials_1.aspx) has been used by myrmecologists for many years to make plaster-bottom nests for housing ant colonies in the laboratory, but other brands of orthodontic plaster are also on the market. All set quickly; working life of the slurry is less than 10–15 min.

**Figure 2.**
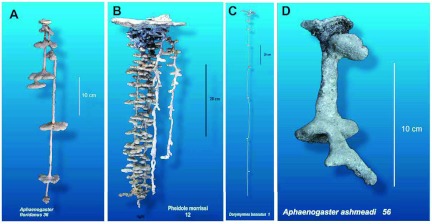
Examples of casts made of different materials. (A) *Aphaenogaster floridana*, dental plaster; (B) *Pheidole morrisi*, aluminum; (C) *Dorymyrmex bossutus*, zinc; (D) *Aphaenogaster ashmeadi*, paraffin wax. High quality figures and videos are available online.

An example of a dental plaster cast is shown in Figure 2A.

**Paraffin wax.** Paraffin wax has the advantages that it requires only easily available equipment and that the entombed ants can be recovered (chamber by chamber, if desired) quickly if the cast is simply melted. Moreover, the ants are usually intact, and brood can be recovered as well (Figure 1B). The disadvantage is that wax is a weak material, so the cast may bend and distort, although careful handling can mitigate this problem. More seriously, molten paraffin is a true liquid and can penetrate into the soil, incorporating soil into the cast. The result is a cast that is more than the interior hollow space. This problem is greater near the surface, where hot wax flows until the nest is filled, heating the upper soil region and allowing wax to penetrate into the soil. It can be reduced by working in wet soils or even heavy watering of the soil before the wax is poured. Alternatively, the soil-containing wax can be carefully scraped away from the cast until soil-free wax is encountered.

Wax supply is as simple as going to the supermarket canning section. Histological wax is somewhat better because it is more pliable. An example of a paraffin cast is shown in Figure 2D.

**Aluminum.** The obvious advantage of aluminum is that it is very light and strong, usually creating a cast that is not broken. The disadvantages are that aluminum casting requires a good deal of equipment and attention to safety and that the ants cannot be recovered from the cast. Metal casts (including zinc casts) are thus excellent for study of architecture and for display but not good for relating architectural features to an ant census. The higher melting point of aluminum (659° C) than of zinc (420° C) dictates that nests with narrow tunnels (in which heat is lost quickly) cannot be readily cast with aluminum. For such nests, zinc is a better choice.

Supply of aluminum is not a large problem, and many potential sources are available. I used aluminum scuba tanks that had failed the pressure test, but they must be cut into pieces small enough to fit into the crucible. An electric hacksaw is useful for this job. Junk yards are a good source of aluminum that can be cut up by hand.

An example of an aluminum cast is shown in Fig. 2B.

**Zinc.** The lower melting point of zinc makes it suitable for nests with much narrower tunnels and finer structure. In fact, zinc will penetrate some distance into shafts as narrow as 1 mm or even less. Its disadvantages are the difficulty of supply, its high density, and its brittleness and its tendency, if heated too much, to ignite and burn, creating a gauzy fluff of zinc oxide that interferes with pouring. Because large casts made with zinc are very heavy, they may break under their own weight, so large casts are better made in aluminum. In theory, zinc should be repairable by soldering, but in practice, I have not succeeded in this endeavor.

An example of a zinc cast is shown in Fig. 2C.

Because zinc is most appropriate for small ant nests, large amounts are not needed. Zinc anodes were used here, but C. Rabeling of the University of Texas, Austin reports (personal communication) that he has successfully used US pennies, which are mostly a zinc alloy. Thirty dollars worth of pennies was more than sufficient for the *Trachymyrmex* nests he was casting.

**Other metals.** Most other metals melt at temperatures too high to be practical and would freeze before penetrating very far into the nest. The only exception is lead, which is not only expensive and toxic but also weak and very dense. It might prove useful for particular applications, such as nests with very fine shafts. Tin is also possible, but like lead, it is very expensive, and no scrap source is available.

### Casting Equipment

**Dental plaster.** All that is needed is a container in which to mix the plaster and from which to pour the slurry and a supply of water for making the plaster and for cleanup.

**Figure 3.**
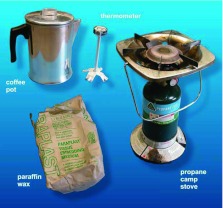
The equipment needed for making nest casts of paraffin wax. The coffeepot makes pouring easy, and the thermometer is needed to prevent overheating of the wax. High quality figures and videos are available online.

**Paraffin wax.** A simple, one-burner, propanefueled camp stove is sufficient for melting the wax in a coffee pot with a pour spout (Figure 3). A thermometer is necessary to keep the temperature of the wax from going above 100° C.

**Aluminum or zinc.** Melting metal in the field is a larger undertaking. It requires some equipment made for the purpose, but making the necessary items is not difficult. Simple physical principles dictate the construction of a kiln and its operation. To the degree possible, the cavity of the kiln should approximate a black-body radiator in the sense that it should be designed to retain within the cavity as much of the heat generated by burning fuel as possible. Burning fuel such as the charcoal used here, requires oxygen, so the kiln must have an air intake. Finally, the molten metal should be contained in a vessel itself resistant to melting, corrosion, and oxidation at the temperatures needed for making effective casts.

The first metal-melting, field “portable” kiln was based on ideas from a booklet purchased from a website (Gingery 2000) that describes how to build a charcoal-fired kiln for making small castings for machine shops. The first kiln was a 20-gallon galvanized garbage can insulated on the inside with an 8–10-cm thick layer of a sand-fire clay mixture; it therefore weighed over 150 kg and was difficult to move to the work site in the field. The draft for this kiln was supplied by a fan scavenged from a car heater from an auto salvage yard (Pick-n-Pull), powered by a marine battery.

In a later version of this kiln a light-weight refractory blanket material (Durablanket, www.infraredheaters.com/insulati.htm) was used to replace the sand-fire clay. It provided an insulation that was at least as effective and much lighter; a single person could easily move the kiln.

**Figure 4.**
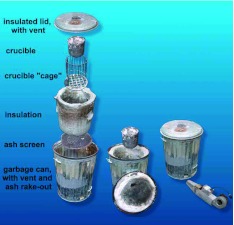
Exploded view of the large forced-draft kiln, showing its parts and their assembly. The air vent at the bottom opens into a space beneath the ash screen, which is supported on a baffle (Figure 5). The insulation and the crucible cage rest on the ash screen, with a space for charcoal between them. Air forced into the bottom rises through the charcoal and is vented through the hole in the insulated lid. High quality figures and videos are available online.

**Figure 5.**
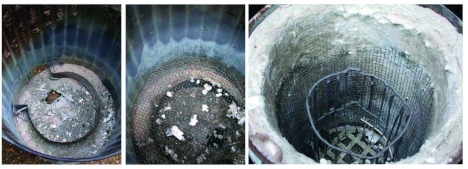
Construction details of the large kiln, (left) The baffle support for the ash screen; (center) the ash screen in place on the baffle; (right) Insulation with its protective screen and the crucible cage in place, ready to receive a load of charcoal and the crucible. High quality figures and videos are available online.

**Figure 6.**
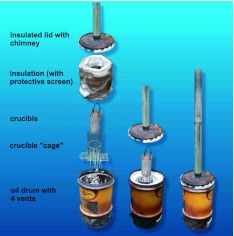
Exploded view showing the construction of the medium, passive-draft kiln. Construction is similar to that of the large kiln, except that 4 air vents were cut at the bottom, and the lid is provided with a tall chimney that creates a draft through the kiln. High quality figures and videos are available online.

**Figure 7.**
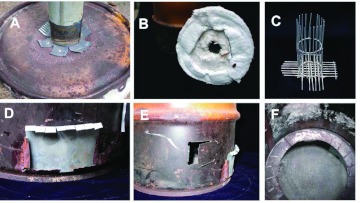
Construction details of the medium kiln. (A) Attachment of the chimney to the lid; (B) underside of the lid, showing insulation; (C) crucible cage and ash screen; (D) ash rake-out; (E) air vents (regulated by bending); (F) lip for support of the crucible cage and ash screen. High quality figures and videos are available online.

**Figure 8.**
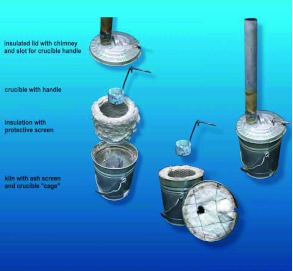
Exploded view of the small kiln, showing its construction. Like the medium kiln, this scaled-down kiln relies on passive draft created by the tall chimney. The crucible is designed for one-handed use and small amounts of molten metal, primarily zinc. The handle projects through a slot in the lid, so that is does not get excessively hot. High quality figures and videos are available online.

**Figure 9.**
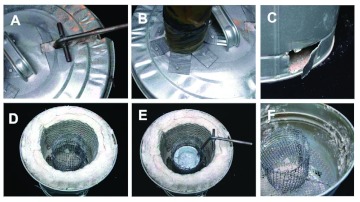
Construction details of the small kiln. (A) the slot for the crucible handle; (B) attachment of the chimney; (C) air vents; (D) insulation, protective screen, and crucible cage in place; (E) same as D but with crucible; (F) insulation removed. High quality figures and videos are available online.

The designs ultimately developed are shown for three sizes of kiln, each suited to certain applications and metals. The construction of each kiln is described below and in Figures 4, 5, 6, 7, 8, 9.

### Kiln construction

#### Construction of a large, forced-draft kiln.

This kiln is suitable for melting up to 6–7 L of aluminum or zinc (Figures 4, 5). It was based on an 80-L garbage can with lid, with an air vent near the bottom to receive the draft from a car-heater fan, powered by a 12-v deep cycle marine battery. A baffle in the bottom of the garbage can supported an ash screen about 10–15 cm off the bottom. Air forced into this bottom space moved up through the charcoal fill and out the vent in the lid. The inner wall of the garbage can was lined with 2–3 layers of the refractory blanket, leaving a space just over one charcoal briquette wide between the blanket and the crucible “cage”, which rested on this ash screen. The cage received the crucible and prevented the charcoal from falling into the void created by the removal of the crucible. The crucible itself was fashioned from the bottom half of a steel scuba tank provided with a stout steel handle attached through holes drilled near the upper rim.

#### Construction of a large, passive-draft kiln.

The size and weight of the forced-draft kiln can be reduced by providing a sufficiently tall chimney. The construction of this kiln (not pictured) is similar to that of the active-draft kiln above, with the following differences. Instead of a single air hole to receive the fan, four evenly spaced air vents were cut near the bottom of the kiln. The metal was left attached by one edge so that it could be bent to regulate air flow. A fitting around the vent was added to the lid that could receive one or two sections of 3”-diameter stove pipe (see the medium passive-draft kiln below). Two sections created a stronger draft than one. When the kiln got too hot, simply removing the lid reduced the oriented draft.

**Construction of a medium, passive-draft kiln.** Scaling everything down produces a smaller kiln that is more suited to casting smaller nests requiring less casting material (Figures 6, 7). The body consisted of a 65 L drum that had contained transmission fluid.

This kiln was designed to create a passive draft, eliminating the need for the fan and battery. Four air vents were cut near the bottom of the drum, and a shelf that resided in the bottom weal was installed to support the ash screen and crucible cage. Enough layers of insulating refractory blanket were installed to leave a space about one charcoal briquette thick between the cage and the blanket. The inner surface of the blanket was protected with a stainless steel screen or expanded steel sheeting. This protective layer tended to corrode and burn and must be replaced at long intervals. The crucible cage for this kiln was constructed from a stainless-steel grid obtained from a junk yard, as was much other material. Obviously, other constructions would work as well. The size of the cage was appropriate for the smaller crucible used in this kiln, constructed from either the bottom part of a steel oxygen bottle or a heavy-walled steel pipe with a bottom welded on. These crucibles held about 1 L of molten metal.

The lower surface of the lid was insulated with refractory blanket, held in place with steel wire. A fitting to receive 3” stove pipe was attached to the upper lid surface around the vent. In operation, one or two sections of stove pipe were fitted to the lid and created the draft needed for efficient and effective combustion of the charcoal.

#### Construction of a small, passive-draft kiln.

Scaling down still more can lead to a kiln easily carried in one hand (Figures 8, 9). The one shown here used a 20-L garbage pail, with four air vents near the bottom, a support for the ash screen and crucible cage, and a fitting on the lid to receive the stove pipe that creates the draft. The kiln was insulated with refractory blanket, with a space about one briquette wide between the blanket and the cage. The inner surface of the blanket was protected with a stainless steel screen.

The crucible for this kiln was fashioned from a short section of heavy-wall steel pipe with a bottom and a handle welded on. Two pouring divots were set at right angles to one another, allowing either forward or lateral pouring. The crucible was designed for small amounts of metal, primarily zinc, and easy handling with a gloved hand.

### Making the cast

**Dental plaster.** To make a cast, first identify the nest to be used, and, if possible, several alternates nearby. For all types of casts, specimens of the ants should be collected for identification. Prepare the nest opening to receive the plaster slurry by clearing away any debris around the entrance, then form the entrance into a funnel shape, which can be done by addition of a berm of soil around the entrance or by careful enlargement of the entrance with a tool. Be careful not to let dirt or debris fall into the entrance that can block it. Such material can be easily removed with a battery-powered vacuum cleaner.

**Figure 10.**
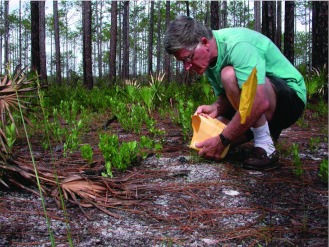
Pouring a thin slurry of dental plaster into a *Formica dolosa* nest. The plaster is best mixed with the hand, so that lumps can be felt and dispersed. High quality figures and videos are available online.

Estimate the amount of plaster needed from experience and the amount of soil around the nest opening. The amount can obviously vary enormously, from a few milliliters to 20 L or more. Use a vessel of appropriate size, preferably one with a spout or pouring lip. Enough plaster should be mixed to fill the nest in a single pour. The purpose of the “alternate nests” is to use up the plaster if too much has been mixed (as is usually the case). Add water to the dry plaster, mixing it with a tool or, better, by hand (which allows detection of lumps). When the mixture has the consistency of thin mayonnaise (i.e., is just capable of being poured), add enough water to make the slurry seem quite thin (20–50% more water), pouring like whole milk. Pour the slurry into the nest opening as fast as possible without overflowing the nest entrance (Figure 10). The reason for the extra water is that the soil will suck water from the slurry as it flows into the tunnels, eventually causing the slurry to become too thick to flow.

When the nest is full, the job is not yet done. As the soil removes water from the slurry, the visible level in the top of the nest will recede. Top it up several times until it no longer recedes.

Working time of the plaster slurry is rather short. By 10–15 min, the slurry will have thickened too much to fill a nest easily. Its life can be extended somewhat by addition of more water, but beyond a certain point, the cast formed with such “extended life” plaster is very weak. Dental plaster sets in less than 30 min, but for additional strength, wait for about an hour before excavating the cast. Excavation methods for all types of casts are discussed below.

**Paraffin wax.** If the wax is melted in an aluminum coffee pot with a spout (Figure 3), it can be easily and accurately poured. As with plaster, wax must be topped up until it sets, although much of the demand will be created by penetration into surrounding soil. Most importantly, use a thermometer to keep the temperature of the molten wax below 100° C. Otherwise the hot wax will cause steam to bubble through the wax, creating froth.

### Operating the kilns

**Figure 11.**
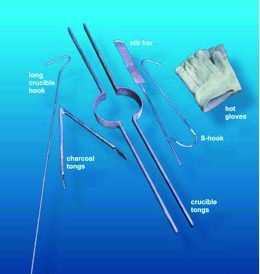
Tools needed for metal casting. Not pictured are a large spoon, a support ring for the crucible after removal from the kiln, and protective face shields. High quality figures and videos are available online.

The operation of all three kilns is largely similar. Tools needed include long-handled tongs for adding charcoal when the kiln is hot, a stirring bar for the molten metal, a large stainless-steel spoon (not shown) for scooping off dross and slag, a stout metal hook about a meter long for fishing the crucible out of the hot kiln, and tongs for carrying the crucible (Figure 11). A metal ring for supporting the round-bottomed crucible after removal from the kiln is also handy (not shown).

**Figure 12.**
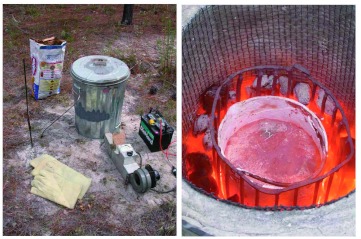
(left) The large kiln in operation. The forced-air draft is provided by a car heater fan powered by a 12-v battery. Stir bar, S-hook, and charcoal are also shown. (right) A crucible containing about 4 L of red-hot aluminum, ready to be poured. High quality figures and videos are available online.

The crucible is placed inside the “cage,” and charcoal is packed into the space between the blanket and the cage. Ignite charcoal before the crucible is added by filling the cage with combustibles such as pine cones, wood kindling, and the like. Once this material has been ignited, turn on the fan (in the active kiln) or cover the kiln with the lid with stove pipe extensions (in the passive kiln) to provide a draft. This technique ignites the charcoal quickly. When the kindling has burned down, the crucible containing the metal to be melted is placed in the cage, and the active or passive draft continued. Properly operated, the large and medium kilns can produce red-hot aluminum, ready to be poured, in an hour or less after ignition (Figure 12; Video 1). As the charcoal burns away, more is added with tongs and hot gloves (burns are a real possibility). The charcoal level should be kept near the level of the lip of the crucible. A crucible that is cooler near the lip will cool the metal, even if the metal is red hot, during the pouring.

When aluminum is used, deeper penetration can be achieved if the metal is heated to well above its melting point. Aluminum melts at dull red heat and is best heated to bright red before pouring (Figure 12, right). When the metal is hot enough, the crucible is extracted from the kiln with a metal S-hook and placed on a metal support ring from which two persons (wearing face masks and gloves) can pick it up using long-handled, home-made crucible tongs (Video 2). The crucible is then carried to the nest, and the aluminum poured into the prepared entrance until the nest is full (Video 3). The aluminum freezes in less than 5 min, so excavation can begin very soon.

**Video 1.**
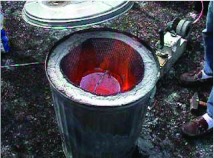
High quality figures and videos are available online.

**Figure 13.**
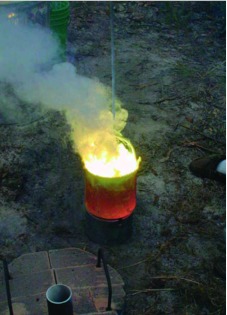
Zinc and zinc slag burn if overheated. The fire can be extinguished by spooning out of the burning material. The chartreuse flame should not be allowed to continue to burn because the fluffy oxides interfere with pouring the zinc. High quality figures and videos are available online.

The small kiln is used primarily for melting zinc, because small ant nests with their narrow shafts are not effectively cast with aluminum or plaster. The metal should be well above the melting point but not hot enough to ignite. This is a difficult point to judge. If the metal ignites, remove the crucible and scoop off the zinc oxide and burning material with a long-handled spoon until the fire goes out (Figure 13).

**Figure 14.**
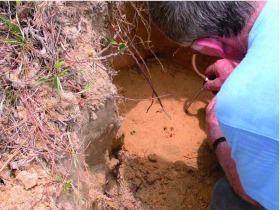
No matter what the casting material, casts are often incomplete. When they are, neat technique pays off, for the opening to the continuation of the nest must be found so that the next stage of casting can be carried out. A vacuum or an aspirator are used to remove soil occluding the nest continuation. High quality figures and videos are available online.

**Incomplete casts.** Commonly the casting material stops flowing before it reaches the bottom of the nest, no matter what material is used. In this case, during excavation, take great care to find the end of the cast and locate a place-holder (a piece of metal or a trowel) where the cast ended. Then, using a trowel and a vacuum, carefully remove soil until the opening of the continuing shaft(s) is found (Figure 14). Fashion a soil funnel around this opening and carry out the next stage of casting. For very deep nests, this process may have to be repeated 3–5 times, and the parts of the casts reassembled later. Working with molten metals in the bottom of a deep pit raises safety issues, including flaming socks. Various long-handled hooks and chains have been developed for lowering and tipping the crucible from the rim of the pit.

### Safety Issues

Obviously, sloshing red-hot molten metals around can be dangerous, and sensible practice is essential. Long-sleeved shirts and long pants should be worn, and during servicing of the kiln or transportation of the hot crucible, full face shields and hot gloves are recommended (Videos 2, 3; for examples of gloves: http://www.artcoinc.com/steelgrip.php). Only once in over 50–60 pours have we experienced any blow-back of liquid metal, and that was when the nest completely surrounded a mass of wet soil. Even then, the spray was only vertical, and we were safe at the ends of the tongs. Nevertheless, the two pourers should have an escape plan in case of blow-back.

Zinc, with its lower melting point, has never caused such problems.

The kiln, when uncovered, radiates intense heat, and can cause burns if one is too close. A red-hot crucible will ignite any dry organic matter within a few centimeters, potentially setting fire to the surroundings. Because those handling the crucible will be occupied, another person should be delegated to put out such fires.

**Video 2.**
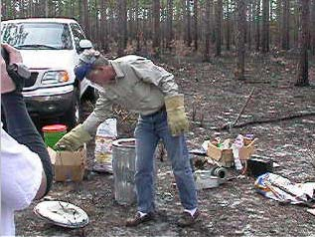
High quality figures and videos are available online.

**Video 3.**
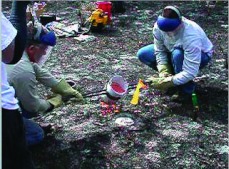
High quality figures and videos are available online.

### Corrosion issues

**Figure 15.**
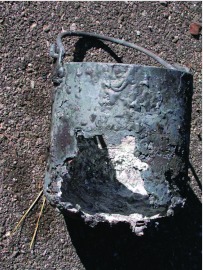
Inattention allowed the kiln to overheat, burning out the crucible and spilling the molten metal into the bottom of the kiln, where it burned a hole and flowed onto the ground underneath. The hole can be seen in Figure 5 (left) but did not affect further operation of the kiln. High quality figures and videos are available online.

Steel crucibles corrode from oxidation of the outside and solution by liquid metal on the inside. The inside of the crucible should be coated with a material that is not wet by liquid metal, such as boron nitride (for example: BN Lubricoat Blue, www.zypcoatings.com/ProductPages/BnCoatings.htm) or some other high temperature coating. The coating must be renewed with every use and greatly reduces corrosion from inside. No coating has yet been discovered for the outside of the crucibles that prevents the spalling due to oxidation of the steel. Very high, run-away temperatures greatly speed the burning of the steel crucibles and can cause them to fail disastrously (Figure 15). As a result of general corrosion, crucibles are good for a limited number of uses and must then be replaced. Fortunately, steel scuba tanks no longer useful for diving are usually available free of charge.

### Excavating the cast

**Figure 16.**
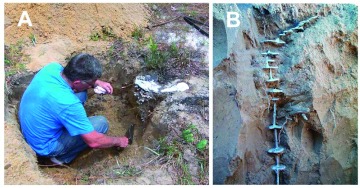
No matter what the casting material, the cast should be excavated from the side, not from above. (A) The pit should be of a depth commensurate with the expected size of the cast. (B) The cast is best exposed for its full depth but left supported by soil until it is ready to be removed. High quality figures and videos are available online.

Excavation proceeds similarly no matter what the casting material. A pit is dug adjacent to the nest, large enough to work in and deep enough to recover the estimated depth of the cast (Figure 16A). The edge of the pit should be 20 to 30 cm from the closest estimated edge of the cast. The edge of the pit is then carefully cut away in the direction of the cast until the cast is encountered. Thereafter, soil is removed around the cast in horizontal layers, gradually exposing the cast to view (Figure 16B). The pit should be deepened as necessary. Leaving the upper portion of the cast enclosed in soil to support it be advantageous while the cast is followed down to its bottom, after which time the entire cast can be freed and removed. If the casting medium is dental plaster, the cast will be removed in pieces, so keeping the pieces separated by level will facilitate later reconstruction. To a lesser degree, the same may also be true of paraffin wax.

### Cast reconstruction

All casts may need some degree of reconstruction. Aluminum can be welded or soldered with only modest equipment, but is not always successfully. Whereas soldering two shafts of similar diameter together is relatively easy, the job gets much harder when the pieces to be soldered are of greatly different size, such as a shaft joining a chamber. Moreover, series 6xxx alloys (such as are used for scuba tanks) are particularly difficult to solder. Solders of varying melting points and hardness are available online. Zinc chloride powder can be used as flux. Support the pieces firmly, coat the parts to be soldered with zinc chloride, heat them with a MAPP gas torch until the flux is molten and glassy, then melt the solder at the joint. Continued heating may be required for the solders with a higher melting point (my preference: www.muggyweld.com/super5.html?OVRAW=welding%20aluminum&OVKEY=aluminum%20welding&OVMTC=standard). These melt slowly and produce the danger of heating the cast itself above the melting point. This must therefore be done carefully.

The pieces of zinc casts can be glued together with grey, slow-hardening epoxy, such as JBWeld, but the cast must be supported on a backboard. I have not succeeded in soldering zinc, although in theory, it should be possible, and have not been able to find a suitable flux.

Paraffin cast pieces can be attached to each other by simple fusion; the ends to be joined are heated above the melting point and held together until the wax hardens.

**Figure 17.**
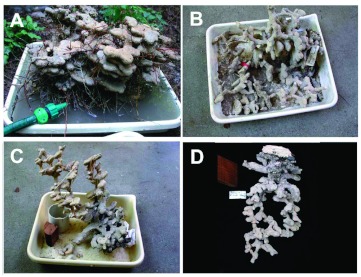
The process of washing a cast, recovering the pieces, drying, and reassembling: (A) Washing away soil; (B) the dry pieces, ready for reassembly; (C) assembly in progress, with 5-min epoxy and with rubber bands for support; (D) the reassembled cast of a *Formica dolosa* nest. High quality figures and videos are available online.

Assembly of dental plaster casts can be tedious and challenging (Figure 17A–D). Five-min epoxy cement works well, only enough for the joints ready for gluing should be mixed. Usually, all joints must be glued one at a time, although one can work on several subassemblies or several casts at once. Finding which pieces join to which others is not as difficult as it may seem. Color of the cast (derived from soil color) gives information about which nest level each piece came from, and by careful inspection and trial and error, one can determine which joints mesh perfectly and should be glued. Plaster is almost never lost during breakage, though pieces may be lost in the soil during excavation; they can be relocated if the soil is sifted.

Larger casts must be supported from a backboard, but smaller ones can be handled and stored without such support.

## Discussion

Understanding ant colonies as superorganisms requires knowledge of the nests they construct and how the ants arrange themselves within them. If nest architecture is regarded as an evolved trait, as it almost certainly is, then the particulars of the architecture must have been produced by natural selection and must contribute to superorganismal fitness. How they do so is largely unknown, but the first step in solving this mystery is certainly to describe the nest architecture of various ant species. Pursuing this line of research requires quantitative descriptions of nest architecture, possibly including such variables as total depth, chamber shape, number, and dimensions, vertical distribution of chambers and their size, number and orientation of connecting shafts, changes in nest “shape” during nest growth, and the number of ants living in the nest and their distribution among the chambers. Comparing species on the basis of these variables might provide insight into how nest architecture evolves and how form might serve function.

The purpose of the present paper is to encourage others to make casts and to study ant nest architecture. Each of the methods presented here is suited for part or all of this job. Metal casting is excellent for making the architecture visible for study and display, dental plaster adds the possibility of recovering the ants, and paraffin wax is excellent not only for rendering the nest architecture but also for capturing all colony members where they were located when the wax was poured. In other words, wax-casting is an excellent tool for capturing entire colonies in their natural spatial arrangement in addition to revealing the architecture of their nest. The spatial distribution of colony members is not random or incidental but is itself an evolved trait serving colony fitness (Sendova-Franks and Franks 1995; Tschinkel 1999).

Other potential casting materials probably exist. For nests with large-diameter tunnels and chambers, plumber's urethane foam shows promise. As an alternative to concrete, this very light-weight material has potential for casting very large nests, though the long hardening time may be a drawback.

An as yet unexplored limitation of nest casting is the effect of soil type on nest architecture. Most of my casts were made in very sandy soil, but the occasional encounter with deeper, denser soil layers suggests that nest architecture is affected by soil “firmness” and “ease of excavation.” When *Pheidole morrisi* Forel (Hymenoptera: Formicidae) dig through a denser soil layer, their chambers become smaller and closer together, but once through this layer, the original size and spacing is resumed. This pattern suggests that some measure of time or effort governs some architectural elements of the nest. This possibility must be tested through colony transplantation into different soil types.

Soil type also creates potential problems for casting. In dense, nonporous soils, the casting material cannot easily displace air from the nest into the surrounding soil, capturing air and causing voids and incomplete casts. Nevertheless, nests as large as those of *Atta bisphaerica* Forel (Hymenoptera: Formicidae) have been successfully filled with concrete (Moreira et al. 2004), although the cast could not be removed because of its weight. Dense, water-saturated soils could potentially also cause blow-back of liquid metal, so casting in such soils should be undertaken carefully.
